# Hydroxychloroquine Improves Obesity-Associated Insulin Resistance and Hepatic Steatosis by Regulating Lipid Metabolism

**DOI:** 10.3389/fphar.2019.00855

**Published:** 2019-08-02

**Authors:** Xin Qiao, Zhuo-Chao Zhou, Rui Niu, Yu-Tong Su, Yue Sun, Hong-Lei Liu, Jia-Lin Teng, Jun-Na Ye, Hui Shi, Cheng-De Yang, Xiao-Bing Cheng

**Affiliations:** ^1^Department of Rheumatology and Immunology, Ruijin Hospital, Shanghai Jiao Tong University School of Medicine, Shanghai, China; ^2^Shanghai Pharmaceutical Medicine, Shanghai, China

**Keywords:** hydroxychloroquine, hepatic steatosis, dyslipidemia, insulin resistance, peroxisome proliferator-activated receptor gamma

## Abstract

The burden of obesity and associated cardiometabolic diseases has been considered as an important risk factor for lupus patients. Therefore, whether obesity is involved in the over-activation of autoimmune response has attracted more and more attention. Hydroxychloroquine is a synthetic antimalarial drug and has been the clinical treatment of rheumatic diseases irreplaceable first-line drugs. Hydroxychloroquine has been suggested to have beneficial effects on lipids and insulin sensitivity, which may contribute in lowering high cardiovascular risk in SLE patients. However, its mechanism on insulin sensitivity and lipid disorders is far from being completely understood. In the present study, the therapeutic effects of hydroxychloroquine were evaluated under pathological conditions *in vivo*. Obesity was induced in C57BL/6 mice fed with high-fed diet, or in mice fed with high-fat diet and hydroxychloroquine. In addition, healthy mice that received normal chow diet were also monitored. The present results revealed that hydroxychloroquine reduced weight, hepatic steatosis, glucose, and insulin resistance. Furthermore, hydroxychloroquine downregulated the expression of peroxisome proliferator-activated receptor gamma in the liver. According to these present results, genes about lipid metabolism went down in high-fat mice liver. Hydroxychloroquine shows potential in ameliorating obesity-induced pathology, which acts though PPARγ to facilitate the healthy function of hepatic tissues. This evidence shows that hydroxychloroquine plays a role in improving obesity-induced lipotoxicity and insulin resistance though the peroxisome proliferator-activated receptor gamma pathway.

## Introduction

For several decades, industrialized countries have been faced with the increasing prevalence of autoimmune-mediated diseases (Pedersen et al., 2007; Patterson et al., 2009). These diseases are the result of complex genetic backgrounds interacting with multiple environmental factors. Genetic factors do not change over time, but the interference of environmental factors is more often found (Bogdanos et al., 2013). Obesity is now a worldwide health problem associated with an increasing number of diseases, including type 2 diabetes, cardiovascular disease, hypertension, and certain cancers. The burden of obesity and associated cardiovascular diseases has been considered as an important risk factor since the 1970s, when Urowitz et al. (1976)displayed a bimodal mortality peak for lupus patients, which ranked only second to infection. Therefore, the involvement of obesity in the pathogenesis of autoimmunity is increasingly linked. It has long been established that obese subjects have subclinical chronic inflammatory states, which can lead to a variety of metabolic disorders. In addition, an increasing number of studies have shown that obesity is significantly associated with higher prevalence or poorer prognosis of many immune diseases (Cao, 2014). The liver plays key roles in glucose and lipoprotein metabolism and is the most important peripheral target tissue of insulin. Νonalcoholic fatty liver disease (NAFLD), which has been characterized as an increase in hepatic triglycerides (TGs) content, is usually found in obese subjects (Matteoni et al., 1999), and a risk factor for many metabolic diseases, including type-2 diabetes mellitus (Lallukka and Yki-Järvinen, 2016) and cardiovascular disease (Targher et al., 2010).

Hydroxychloroquine (HCQ) is one of the antimalarial agent family members and its effectiveness in treatment of rheumatic diseases was recognized during World War II (Wallace, 1996). To date, it is still widely used to treat a variety of autoimmune diseases, such as systemic lupus erythematosus (SLE). It has been proved that HCQ can play its role in immune regulation through a variety of mechanisms, including inhibition of autophagy, antigen presentation, and cytokine chemotaxis. In addition, it reduces the production of pro-inflammatory cytokines, inhibits matrix metalloproteinases, blocks T and B cell receptors, and participates in toll-like receptor signaling (Belizna, 2015; Kaplan et al., 2016). Further, HCQ has been found to improve blood lipid and glucose levels, which may help reduce the high cardiovascular risk in SLE patients (Maiorino et al., 2017). Clinical studies have shown that the application of HCQ significantly decreased SLE with atherosclerosis in the lipid spectrums, including surface cholesterol and TGs. However, it did not increase the occurrence of adverse reactions, but had good safety (Babary et al., 2018). Furthermore, the mechanism of HCQ on insulin sensitivity and lipid disorders is not being completely verified until now.

In the present study, the effect of physiological HCQ on insulin sensitivity in mice fed with high-fat diet (HFD) was examined, and the possible mechanisms were investigated. It was proven that HCQ remitted hepatic steatosis and enhanced insulin sensitivity in the mouse model through decreased adipogenesis, as well as peroxisome proliferator-activated receptor gamma (PPARγ), sterol regulatory element-binding transcription factor 1 (SREBP1c), and carbohydrate response element binding protein (ChREBP) expression in liver tissues of obese mice. Based on these results, it was suggested that this compound might downregulate adipogenesis to decrease hepatic steatosis that had a protective effect on insulin resistance and systemic inflammation in obese mice.

## Materials and Methods

### Animal Treatment

This study was carried out in accordance with the principles of the Basel Declaration and recommendations of International Association of Veterinary Editors guidelines, Laboratory Animal Ethics Committee of Ruijin Hospital. The protocol was approved by the Laboratory Animal Ethics Committee of Ruijin Hospital.

Six-week-old male C57BL/6J mice (Shanghai SLAC Laboratory Animal Co., Shanghai, China), weighing approximately 18 g, were individually housed in cages at a temperature of 23 ± 2°C with a humidity condition of 55 ± 5% and standard light (12-h light/dark) cycle.

**Figure 1:**
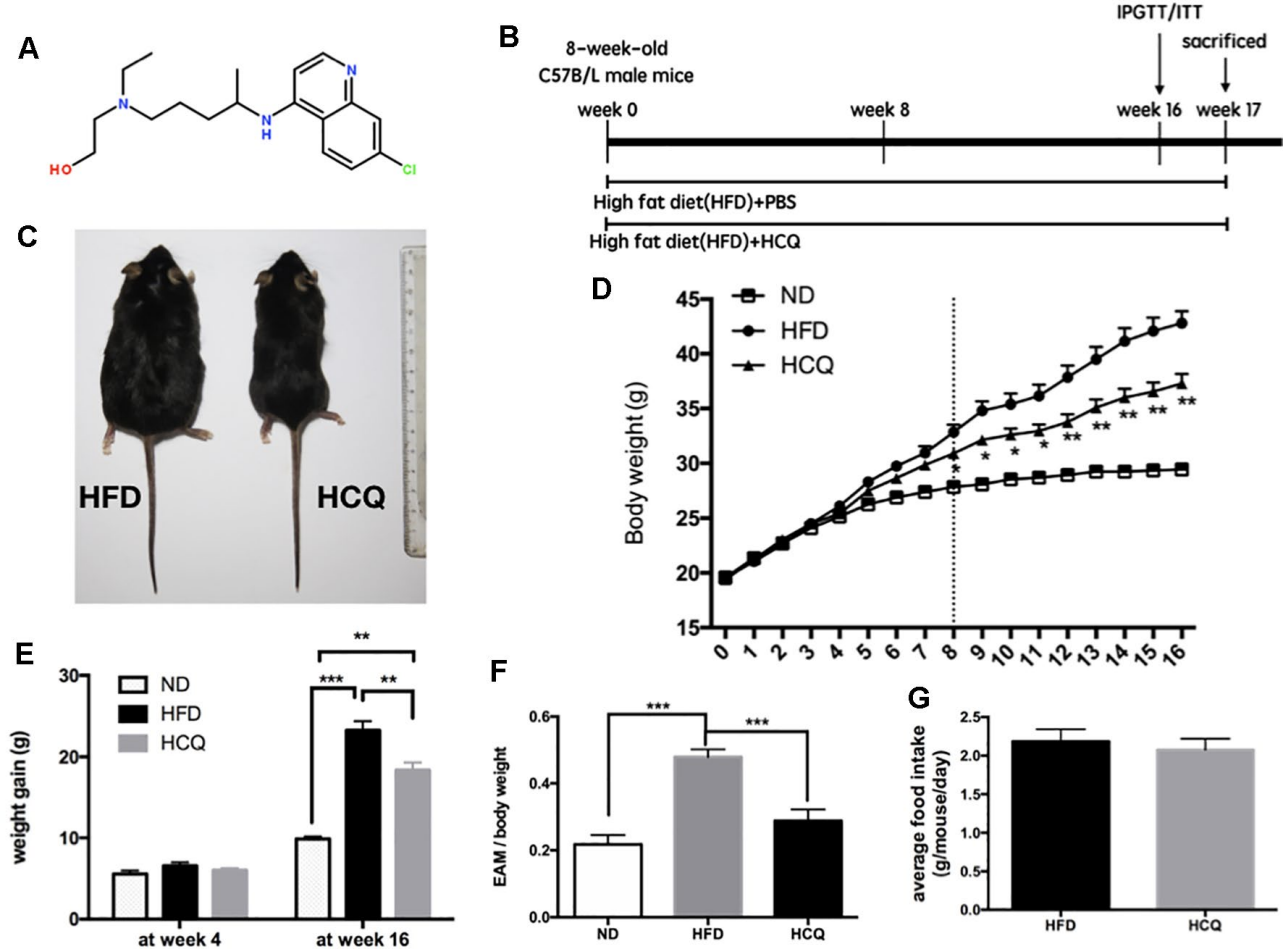
Effects of hydroxychloroquine (HCQ) treatment on body weight and fat mass in HFD-induced obese mice. **(A)** Chemical structure of HCQ. Its molecular formula is C_18_H_26_ClN_3_O. Molecular weight (MW): 335.872 g/mol. **(B)** Timeline and scheme of the in vivo experiment. Eight-week-old male C57BL/6 mice were given high-fat diet (HFD) with or without HCQ [1 mM, 40 mg/kg of body weight (BW)/day] by feeding mice ad libitum for 16 weeks. After 16 weeks, all mice were subjected to the glucose tolerance test or test for insulin tolerance (n = 12) and allowed to recover for 1 week. Then, liver tissue and serum were harvested for biochemical analysis. **(C)** Representative pictures of mice at end of the experiment, scale bar = 1 cm. **(D)** Growth curve and **(E)** body weight gain of mice in the normal diet (ND), HFD, and HFD + HCQ groups after 4 and 16 weeks. Ten samples were taken per group for each assay. **(F)** The epididymisadipose mass (EAM) of body weight in each group at 16 weeks. **(G)** The average food intake. Data were presented as mean ± standard error of the mean (SEM) in each group. Significant difference: *P < 0.05, **P < 0.01 vs. the ND or HFD group.

After 2 weeks of adaptation to our facilities, 36 mice (8-week-old) were randomly divided into three groups: normal chow diet (ND) group, HFD group, and HFD pre-treatment with HCQ group. Mice in the HFD group were given a HFD (60% calories from fat, 20% calories from protein, and 20% calories from carbohydrate; Research Diets) for 17 weeks (25-week-old). Mice in HCQ group were given a HFD pre-administered with 1 mM HCQ (sodium HCQ in water, 40 mg/kg/d) for 17 weeks (**Figure 1B**, time point 0). Mice in the ND group were merely given normal chow diet. Body weight, food intake, and fasting blood glucose (FBG) level were recorded weekly. Intraperitoneal glucose tolerance test (IPGTT) and insulin tolerance test (ITT) were tested at week 16 of treatment. During week 17 of treatment, blood samples were collected and related parameters were detected using the appropriate kits, following the instruction of the manufacturer. The liver tissue and epididymal visceral adipose tissue were dissected, weighed, and frozen in liquid nitrogen immediately and stored at −80°C.

### Determination of Glucose Homeostasis

In order to evaluate glucose and insulin tolerance, IPGTT and ITT were performed. Mice were fasted for 12 and 4 h separately, and blood samples were collected from the tail for baseline of glucose level. Then, each animal was injected intraperitoneally with glucose (Sigma, 1 g/kg body weight) or insulin (Novolin R, 0.75 U/kg body weight). Afterwards, blood samples were collected from the tail at 15, 30, 60, and 120 min after injection to immediately analyze glucose concentration.

The area under the curve (AUC) was calculated using SigmaPlot 11.0 (Systat Software Inc., San Jose, CA, USA). Blood samples at 0, 15, and 30 min were also used to determine the insulin level based on a previously described method (Wallace et al., 2004). The homeostasis model assessment (HOMA) was calculated using a HOMA2 calculator (Folch et al., 1957).

### Biochemical Analysis

At the end of the animal treatment, blood samples were harvested and serum was separated for estimation of total cholesterol (TC), low-density lipoprotein-cholesterol (LDL-c), high-density lipoprotein-cholesterol (HDL-c), TG, and free fatty acids (FFAs) using commercial kits (Shanghai Kehua Bio-Engineering Co., Ltd). The assays were conducted according to the manufacturer’s instructions.

Liver TG and TC content were measured following Folch extraction (Sun et al., 2011). Then, the dried lipid residues were resuspended in 800 μl of ethanol with 1% triton for TC and TG assays. Liver TC and TG levels were examined using the same kit used in the plasma analysis.

Serum samples were collected for IL-1β, TNF-α, and IL-6 analyses. Measurements were performed using enzyme-linked immunosorbent assay (ELISA) kits (Anogen, Toronto, Canada), according to the manufacturer’s instructions.

### Quantitative RT-PCR

Total RNA was isolated from cells or homogenized tissues using TRIzol reagent (Invitrogen). One microgram of total RNA was reverse transcribed using PrimeScript Reverse Transcriptase (Takara). The resulting cDNAs were amplified using 2× SYBR Green qPCR Master Mix (Takara), and the gene expression was analyzed by quantitative real-time PCR with the LightCycler 480 System (Roche, Germany) using the SYBR Premix. The primer sequence details are presented in **Table 1**, and the relative mRNA levels were calculated and normalized to glyceraldehyde phosphate dehydrogenase (GAPDH).

**Table 1:** Primers used for Qt-PCR analysis of gene expression.

	Forward	Reverse
**PPAR**γ	5′-GGAGCCTAAGTTTGAGTTTGCTGTG-3′	5′-TGCAGCAGGTTGTCTTGGATG-3′
**Mgat1**	5′-TGGTGCCAGTTTGGTTCCAG-3′	5′-TGCTCTGAGGTCGGGTTCA-3′
**CPT1**α	5′-CTATGCGCTACTCGCTGAAGG-3′	5′GGCTTTCGACCCGAGAAGA-3′
**CPT1**β	5′-CTCCTGGAAGAAACGCCTTATT-3′	5′-CACCTTGCAGTAGTTGGAACC-3′
**SREBP1c**	5′-GATGTGCGAACTGGACACAG-3′	5′-CATAGGGGGCGTCAAACAG-3′
**ChREBP**	5′-GATCCGACACTCACCCACC-3′	5′-TTGTCCCGGCATAGCAACTTG-3′
**FAS**	5′-GGAGGTGGTGATAGCCGGTAT-3′	5′-TGGGTAATCCATAGAGCCCAG-3′
**ACC**	5′-AGGAGGGAAAGGGATCAGAA-3′	5′-TGTGCTGCAGGAAGATTGAC-3′
**CD68**	5′-CCATCCTTCACGATGACACCT-3′	5′-GGCAGGGTTATGAGTGACAGTT-3′
**Mcp1**	5′-ACTGAAGCCAGCTCTCTCTTCCTC-3′	5′-TTCCTTCTTGGGGTCAGCACAGAC-3′
**IL-1**β	5′-GAAATGCCACCTTTTGACAGTG-3′	5′-TGGATGCTCTCATCAGGACAG-3′
**TNF-**α	5′-CAGGCGGTGCCTATGTCTC-3′	5′CGATCACCCCGAAGTTCAGTAG-3′
**Arg-1**	5′-TGTCCCTAATGACAGCTCCTT-3′	5′-GCATCCACCCAAATGACACAT-3′
**GAPDH**	5′-CTGCCCAGAACATCATCCCT-3′	5′-GGTGGAAGAGTGGGAGTTGC-3′

### Western Blot Analysis

To analyze protein, the collected cells or liver tissue homogenates were suspended in RIPA buffer containing a protease inhibitor cocktail (Roche, Switzerland) and PMSF (Beyotime, China). Then, same amounts of protein were loaded and separated by 10% SDS-PAGE and transferred onto a polyvinylidene difluoride membrane (Millipore, USA). The samples were blotted overnight using related antibodies against PPARγ, SREBP1c, and ChREBP (Proteintech, Wuhan, Hubei, China), fatty acid synthetase (FAS, Proteintech, China), acetyl-coa carboxylase (ACC, Santa Cruz, USA), and phosphorylated serine/threonine-specific protein kinase (p-AKT), total serine/threonine-specific protein kinase (t-AKT), phosphorylated insulin receptor substrate 1 (p-IRS1), total insulin receptor substrate 1 (t-IRS1), and GAPDH (Proteintech, China). These experiments were repeated three times. The AlphaEascFC system (FluorChem 8800) was used for protein quantification.

### Histology

For histological analyses, the livers were isolated and fixed in 4% paraformaldehyde solution for 24 h. Then, the slides were counter-stained with hematoxylin for 10 min and dehydrated in ethanol. Afterwards, the tissues were embedded in paraffin, cut into 5-μm sections, and stained with hematoxylin and eosin (H&E) to assess for histopathological changes.

### Glucose Uptake in the HepG2 Cell Line

HepG2 cells were incubated in DMEM containing 10% fetal bovine serum medium with low glucose for 12 h. Subsequently, these were incubated in serum-free medium DMEM with low glucose for 12 h and finally in 100 nM insulin and 10% fetal bovine serum with high glucose DMEM medium intervention for 24 h. Then, the cell model of insulin resistance was established. Next, cells were incubated with or without HCQ (0, 0.1, 1.0, and 10.0 μM) for 12, 24, 36, 48, and 60 h. Then, glucose uptake was measured using a Glucose Oxidase kit and Enzychrom Glycerol Assay kit, according to the manufacturer’s protocols.

### Statistical Analysis

Data were presented as mean ± standard error of the mean (SEM) from multiple samples. All experiments were repeated at least three times. Group differences were considered to be statistically significant at *P* < 0.05, as assessed by Student’s t-test or one-way ANOVA, followed by the Newman–Keuls multiple-comparison *post hoc* test using Graph-Pad Prism software.

## Results

### Hydroxychloroquine Protected Mice From HFD-Induced Obesity

HCQ was for the first time synthesized in 1944, and the chemical structure of the compound HCQ is presented in **Figure 1A**. It has been demonstrated by several studies that synthesis of lipid is correlated to high serum glucose levels, insulin resistance, and pathogenesis of diabetes (Winzell and Ahren, 2004; Kusminski et al., 2016). An HFD mouse model was used to assess the influence of HCQ on the synthesis of lipid and related disorders. The timeline of HCQ treatment of mice is presented in **Figure 1B**. Eight-week-old C57BL/6 mice were fed only with HFD for 17 weeks to induce obesity as control mice, while the group of treated mice were given intraperitoneal injection (40 mg/kg/day) of 1 mM HCQ and HFD for 17 weeks. The results in **Figure 1** reveal a significant difference in size between the processed and unprocessed mice (**Figure 1C**), and a much slower weight gain in mice treated with HCQ starting from the 6^th^ week (**Figure 1D**). At the end of the 16-week treatment, the average body weight gain of mice in the HFD group reached 23.25 g, while the weight gain in the HFD + HCQ group was significantly lower, with a value of 18.35 g (**Figure 1E**). In addition, the mean fat mass of mice in the HFD and HFD + HCQ groups at week 16 reached 47.9% and 28.8%, respectively (**Figure 1F**). The results in **Figure 1G** show that HCQ treatment did not impact appetite when compared with controls. These results indicate that HFD effectively induced obesity and HCQ reduced body weight and fat mass in HFD-induced obese mice.

### Hydroxychloroquine Prevented Diet-Induced Insulin Resistance and Improved Glucose Homeostasis in HFD-Fed Mice

**Figure 2:**
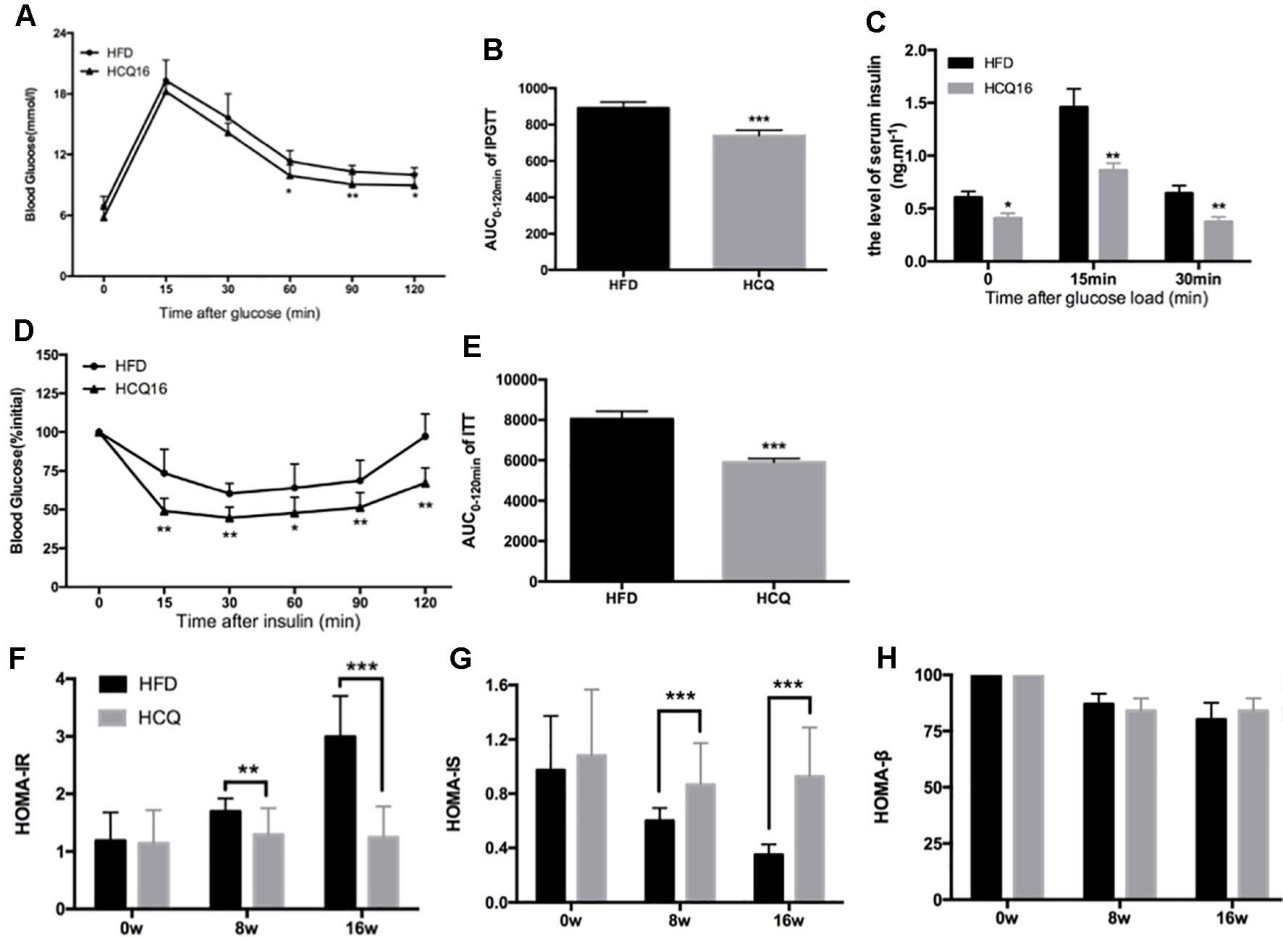
HCQ improved glucose homeostasis and insulin sensitivity in HFD-fed mice. **(A)** The i.p. glucose tolerance test on HDF mice treated by injecting 0.75 g of glucose per kg after overnight fasting. **(C)** Serum insulin concentration at 0-min (fasted overnight) and after glucose load at 15 and 30 min during the i.p. glucose tolerance test. **(D)** The insulin tolerance test was evaluated throughout the study, and 1 U of insulin per kg was injected after 6 h of fasting. **(B** and **E)** The AUC of the IPGTT and ITT is shown. **(F)** The homeostasis model assessment-estimated insulin resistance (HOMA-IR). **(G)** The insulin sensitive index (HOMA-IS). **(H)** The basic function of pancreatic β cell (HOMA-β). Values were presented as mean ± standard error of the mean (SEM). The error bars represent the SEM (n = 8–10). Significant differences compared with HFD group controls were indicated by *P < 0.05, **P < 0.01, and ***P < 0.001 (assessed by Student’s t-test).

Mice fed with HFD developed insulin resistance and raised blood glucose (Qiu et al., 2017). The effects of HCQ on glucose tolerance and insulin sensitivity were determined by IPGTT, ITT, and insulin release test (IRT). The results in **Figures 2A, B** show that the mice treated with HFD + HCQ had a better range of blood glucose fluctuations than those only with HDF, and the blood glucose recovered more rapidly after glucose injection. Moreover, basal fasting plasma insulin levels were similar between these two groups of mice. The IRT results revealed that HCQ-treated mice exhibited significantly higher plasma insulin levels at 15 min after glucose administration (**Figure 2C**). Next, ITT was used to determine if the enhanced glucose uptake capacity of HCQ-processed mice was correlated to insulin use (**Figures 2D, E**). These data showed that HCQ-processed animals had lower insulin resistance, when compared with unprocessed HFD mice. HOMA was put into use to assess the level of insulin resistance, including the insulin resistance index (HOMA-IR), insulin sensitive index (HOMA-IS), and basic function of pancreatic β cell (HOMA-β) (Chakrabarti et al., 2009). As demonstrated in **Figures 2F–H**, HCQ strongly weakened the HFD-induced increase of HOMA-IR and declined in HOMA-IS. However, HCQ had no affection in HOMA-β.

### Hydroxychloroquine Blocks HFD-Induced Hepatic Steatosis With Significant Effect on Serum Lipid Levels

**Figure 3:**
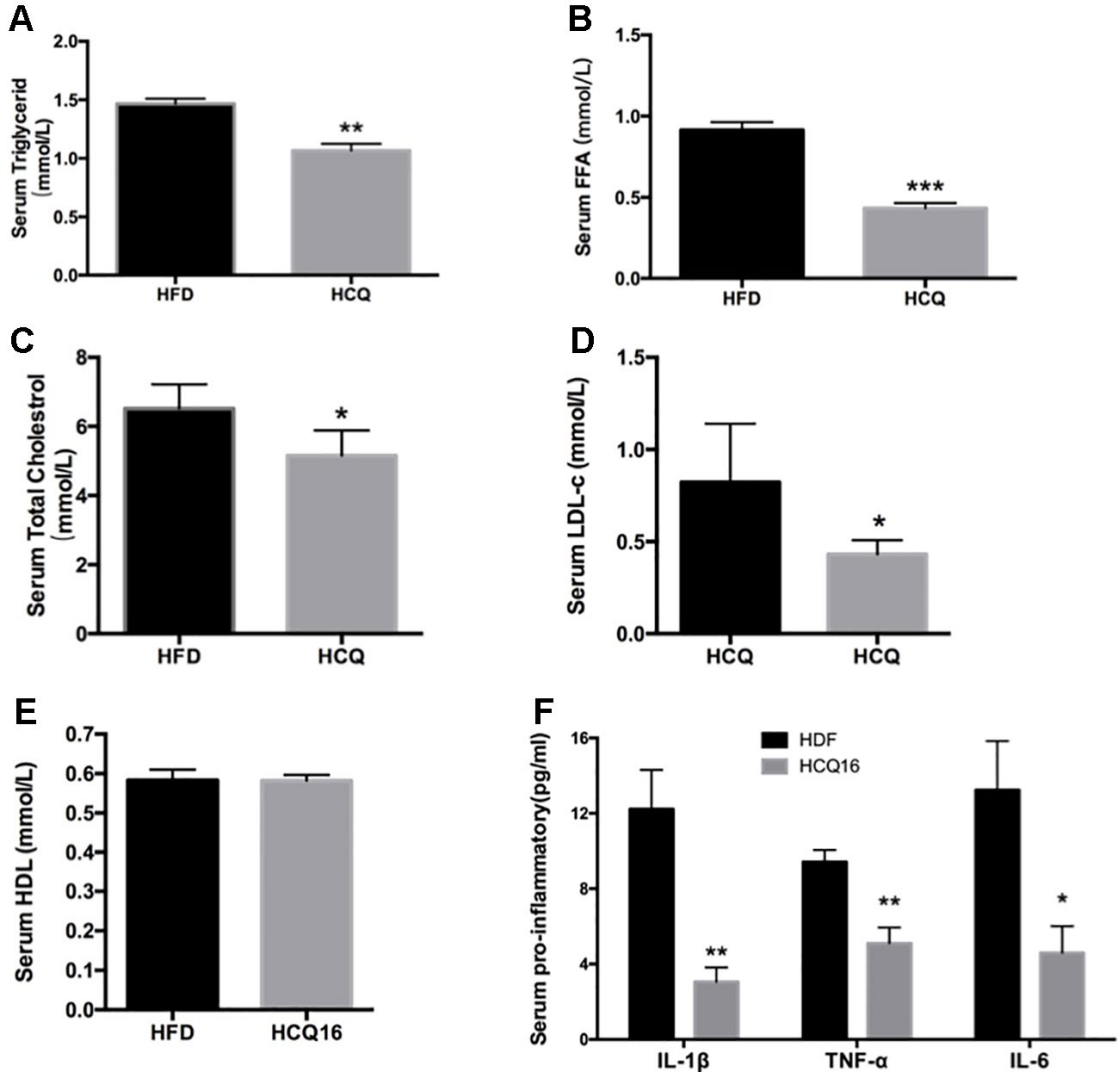
HCQ reduces lipids in serum of high-fat induced mice. **(A)** (FFA), n = 11. **(B)** Triglyceride (TG), n = 8. **(C)** Total cholesterol (TC), n = 6. **(D)** Low-density lipoprotein cholesterol (LDL-c), n = 9. **(E)** High-density lipoprotein cholesterol (HDL-c), n = 11. **(F)** The pro-inflammatory cytokines IL-1β, ΙL-6, and TNF-α, n = 5. After overnight fasting, the levels were measured in the serum of mice treated with vehicle or HCQ for 16 weeks. The values were presented as mean ± standard error of the mean (SEM). The error bars represent the SEM. Significant differences compared with vehicle controls were indicated by *P < 0.05 and **P < 0.01 (assessed by Student’s t-test).

The treatment with HCQ significantly reduced FFA, TG, TC, and LDL-C mean levels by 52.6%, 27.1%, 20.9%, and 47.4%, respectively, when compared with the HFD group. However, this had no effect on HDL-C content, when compared with HFD mice. In addition, the levels of pro-inflammatory cytokines, including TNF-α, IL-1β, and IL-6, in the serum of HFD mice were also examined (**Figure 3**). These results demonstrate that HCQ markedly reduced serum lipid levels and improved the disorders of lipid metabolism and chronic inflammatory state.

**Figure 4:**
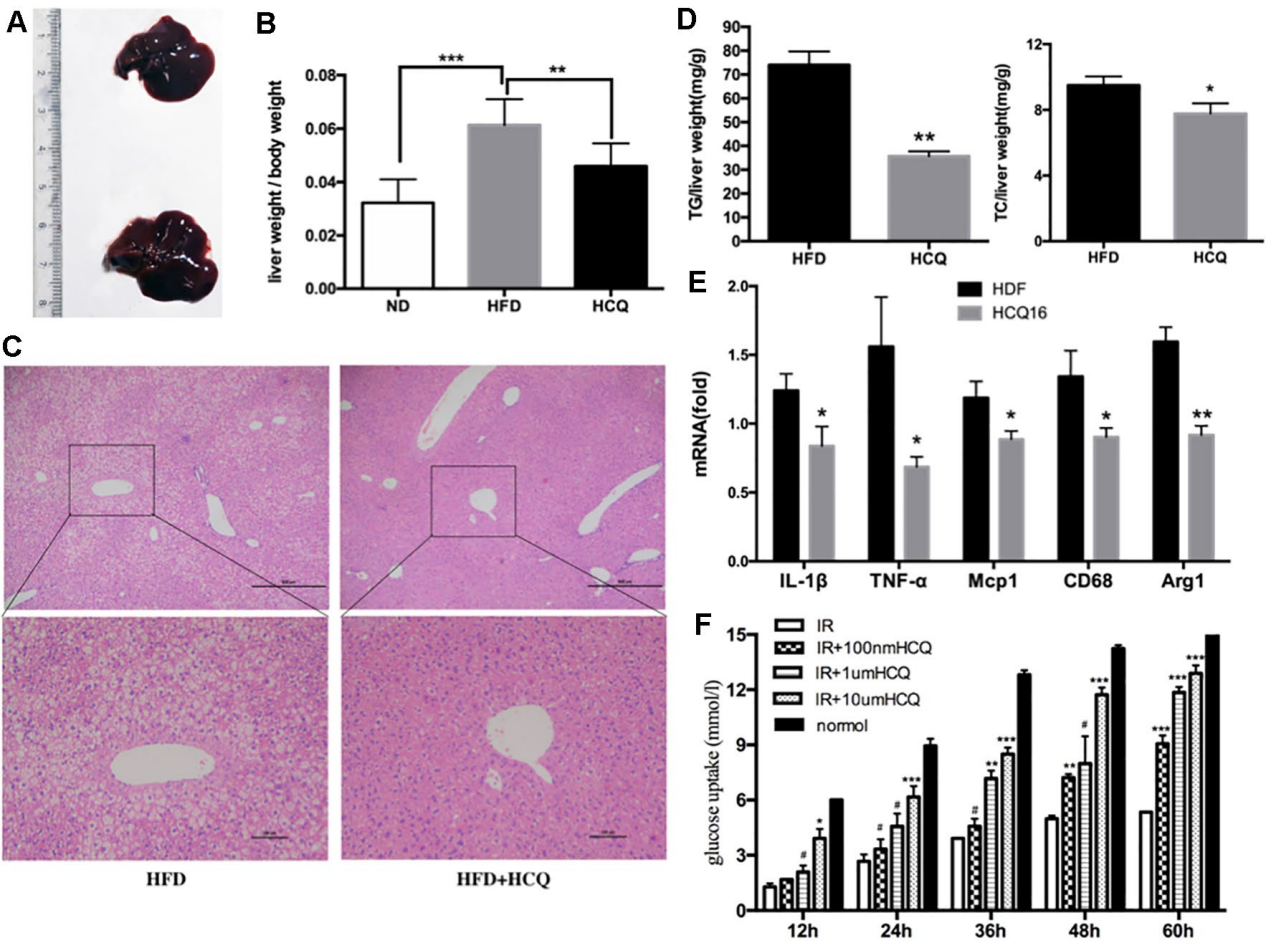
HCQ improved HFD-induced hepaticectopic lipid accumulation. **(A)** Liver tissues were extracted from HFD mice and HFD mice treated with HCQ. **(B)** The liver weight per body weight. Liver section paraffin-embedded samples were analyzed for histopathological changes after staining with hematoxylin–eosin. **(C)** Scale bar = 200 μm and 100 μm. **(D)** The levels of triglyceride (TG, n = 8) and total cholesterol (TC, n = 6) measured in the liver of mice treated with vehicle or HCQ for 16 weeks. **(E)** The mRNA levels of pro-inflammatory cytokines in hepatic tissue were analyzed by real-time PCR. **(F)** The glucose uptake in HepG2 cells incubated by 0.1, 1.0, and 10.0 μM of HCQ for 12, 24, 36, 48, and 60 h. The values were presented mean ± standard error of the mean (SEM). The error bars represent the SEM. The significant differences compared with vehicle controls were indicated by *P < 0.05 and **P < 0.01 (assessed by Student’s t-test).

Liver is one of the most important target organs of insulin. In order to investigate the effects of HCQ on liver fat accumulation, we measured the liver lipid levels of mice in the experimental group and the control group. It was found that the liver fat content of the control group significantly increased, and the liver volume and weight were also larger (**Figures 4A, B**). Furthermore, hematoxylin–eosin staining revealed that HCQ-processed mice had less fat integrant when compared to HFD mice (**Figure 4C**). In addition, hepatic steatosis was investigated by determining the amount of TG and TC in the liver. Consistent with the histological data, it was found that the level of both TG and TC was lower in HCQ-treated mice (**Figure 4D**). Next, the mRNA levels of pro-inflammatory cytokines, including TNF-α, Mag-1, IL-1β, CD68, and Arg-1, in liver tissues of HFD mice were examined. All of these were found to be lower in HCQ-treated HFD mice (**Figure 4E**). Furthermore, HCQ markedly promoted glucose uptake in HepG2 cells (**Figure 4F**). Taken together, the present *in vivo* results advised that HCQ decreased the mRNA levels of pro-inflammatory cytokines and the plasma lipid levels and prevented the hepatic steatosis induced by HFD.

### Hydroxychloroquine Attenuates the Insulin Signaling Pathway in Liver Tissues

**Figure 5:**
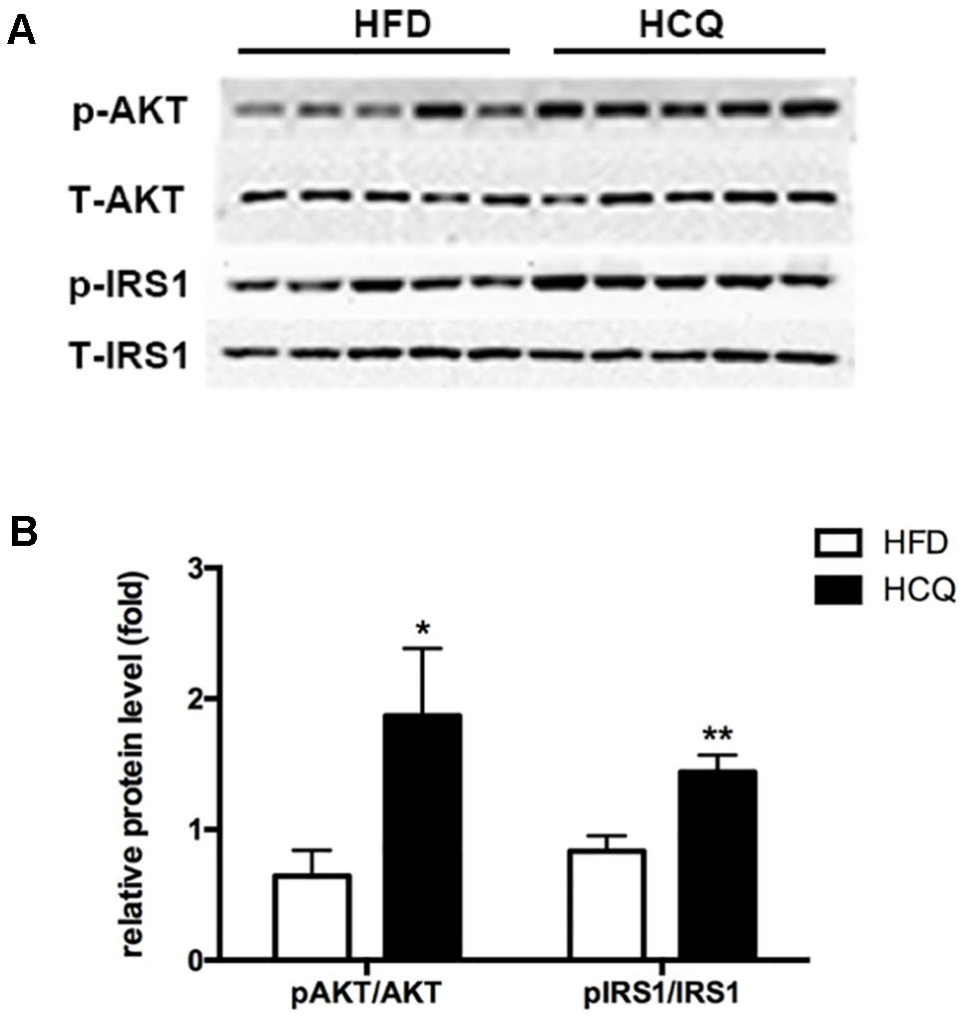
Effects of HCQ on the HFD-induced insulin signal pathway. The phosphorylation of IRS-1 (Tyr896) and Akt (Ser473) were markedly increased by HCQ in HFD-induced mice. **(A)** (f) Representative western blots, **(B)** Grayscale statistical map. Data were presented as mean ± standard error of the mean (SEM) in the HFD and HCQ groups. Significant difference: *P < 0.05 and **P < 0.01 vs. the HFD group.

The phosphorylation of Akt and insulin receptor subsrate-1 (IRS-1) are critical mediators of the insulin signaling pathway (Kitada et al., 2016). In order to investigate the molecular mechanism by which HCQ enhances insulin resistance, the activation of Akt and IRS-1 was detected in liver tissue. As shown in **Figure 5**, the phosphorylation of Akt (Ser473) and IRS-1 (Tyr896) was markedly raised by HCQ. These data suggest that HCQ can prevent the occurrence of abnormal glucose tolerance and hyperinsulinemia induced by HFD and protect insulin sensitivity.

### Effects of Hydroxychloroquine on mRNA and Protein Expression Associated With Lipogenesis in Hepatic Tissue

**Figure 6:**
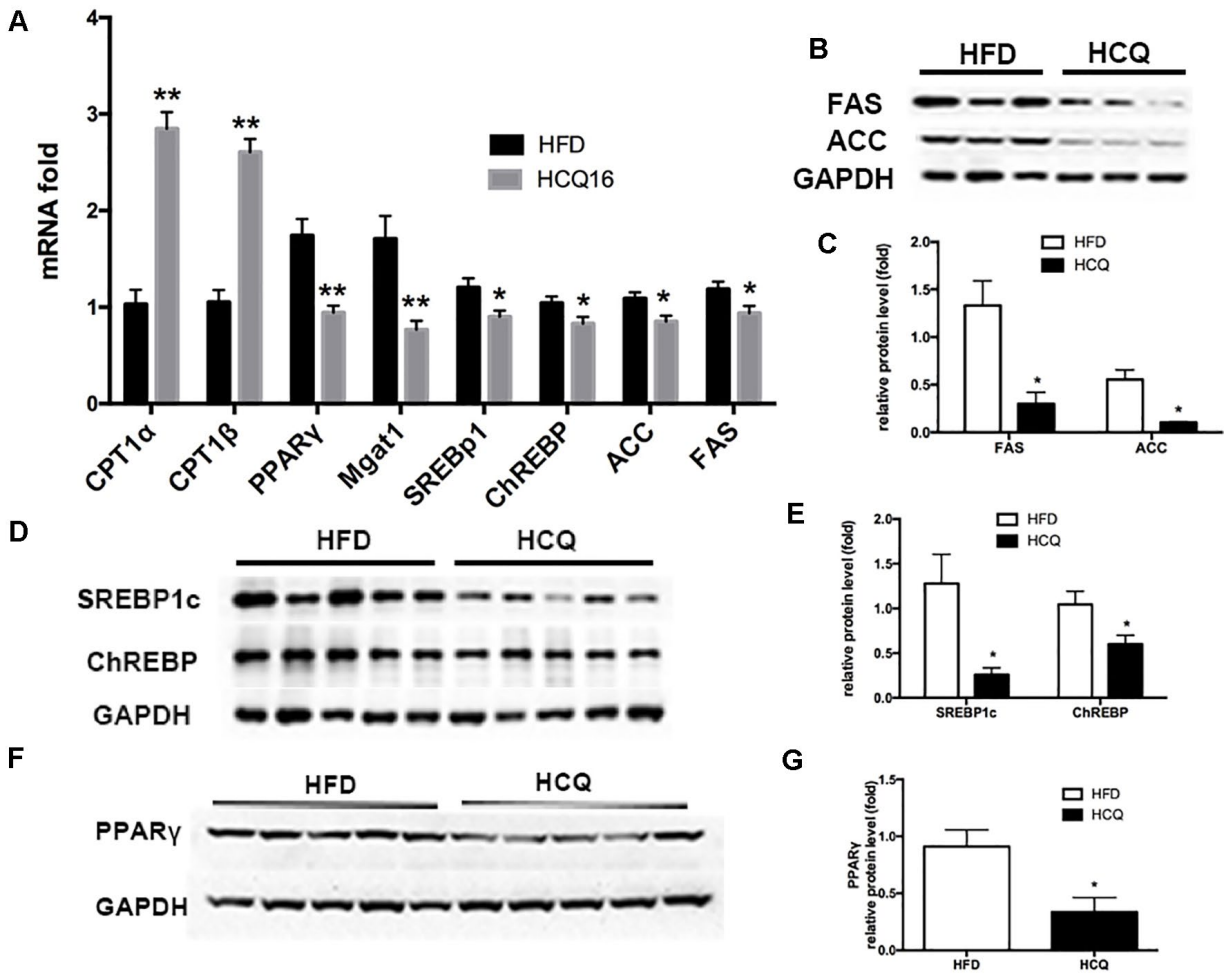
HCQ regulated mRNA and protein expression in liver tissues. The **(A)** mRNA and **(B–G)** protein expression pertaining to lipometabolism as CPT1α, CPT1β, SREBP1c, ChREBP, FASN, ACC1, Mgat1, and PPARγ. Data are presented as mean ± standard error of the mean (SEM) in the HFD and HFD + HCQ groups. Significant difference: *P < 0.05 and **P < 0.01 vs. the HFD group.

As shown in **Figure 6**, mRNA expression of genes involved in lipogenesis (SREBp1c and ChREBP), lipid enzymes (FAS and ACC), and beta-oxidation (CPT1α and CPT1β) was downregulated in the HCQ-treated group when compared to the HFD group (**Figure 6A**). Furthermore, protein expression of adipogenesis was consistent with the mRNA expression level (**Figures 6B–E**). These results indicate that HCQ inhibited the *de novo* lipogenesis (DNL) of liver tissue. In order to further study HCQ’s influence on the molecular mechanism of liver fat content, we tested the PPARγ mRNA and protein level and its target monoacylglycerol O-acyltransferase (Mgat-1) in mice (**Figures 6A, F, G**). Overall, these results indicate that HCQ stimulated the lipogenesis of hepatic tissues by inhibiting the expression of FAS and ACC. In addition, HCQ promoted glucose uptake and enhanced insulin sensitivity by downregulating the PPARγ level.

## Discussion

Cardiovascular morbidity and mortality remain a challenge in the management of patients with SLE. The higher risk of cardiovascular disease in SLE patients is mostly correlated to accelerated atherosclerosis. This process is driven through the complex reciprocity between atherosclerosis pathogenesis and SLE. It has been confirmed that obesity is one of the pathogeneses of many metabolic abnormalities, including cardiovascular abnormalities such as atherosclerosis, and the pathogenesis of many metabolic abnormalities, including cardiovascular abnormalities such as atherosclerosis. Insulin resistance has been demonstrated as a crucial factor that contacts metabolic diseases with obesity (Donath, 2016). There is growing evidence that metabolic disorders are associated with immune disorders (Giannelou and Mavragani, 2017). Pathologically, inhibition of the TNF or IL-1β pathways may benefit patients with diabetes. But the use of immunomodulatory drugs to ameliorate metabolic disorders and associated cardiovascular risks needs to be further investigated. Until now, the medicines that have found stronger effect on glycolipid control are the oldest ones, such as salsalate and HCQ. HCQ is a cornerstone in treating SLE patients and has been considered to exert a broad spectrum of beneficial effects on disease activity and traditional cardiovascular risk factors, such as dyslipidemia and diabetes (Boden, 2011). Cheap and old medications could be a cost-efficient and useful choice in the treatment of individuals with metabolic disorders in SLE. However, it remains indistinct how HCQ might regulate metabolism disorders. In the present study, the effect of HCQ on anti-obesity and glycolipid metabolism was investigated, and the underlying mechanisms were explored. The major findings were that HCQ could significantly reduce fat mass, glucose level, blood serum lipid profiles, and body weight and improve insulin resistance caused by obesity, indicating that HCQ acts on regulating glycolipid metabolism in HFD-induced obese C57BL/6J mice.

Among all immunomodulatory drugs, the one with the most beneficial data on metabolism is HCQ, which has been tested for the longest time and has the largest number of patients so far. Moreover, none of these animal experiments have been conducted, in which the oral administration of HCQ significantly improved insulin resistance in HFD-induced diabetic rats and mice. The present study demonstrated that HCQ not only exerted an anti-obesity effect (**Figure 1**) but also reduced blood glucose levels (**Figure 2**), serum lipids (**Figure 3**), and hepatic steatosis (**Figure 4**) in HFD-induced obese mice. Consistent with the present ITT and IPGTT data, the level of phosphorylated AKT and IRS-1 increased in liver tissues obtained from HCQ-processed HFD mice (**Figure 5**).

Lipid metabolic disorders stimulate the excessive production of FFA in obese individuals, which decreases insulin-stimulated glucose uptake in the whole body (Takamura et al., 2012; Frohnert et al., 2013). In the present study, it was found that HCQ reduced FFA and TG levels more than TC levels after HCQ administration in the HFD + HCQ group. In addition, LDL-c markedly decreased, but there was no effect on HDL-c in the HFD + HCQ group (**Figure 3**). These results suggest that HCQ has a certain effect on regulating lipid metabolism dysfunction and beneficence for accelerated atherosclerosis.

Abnormally high FFA can also lead to glucose disruption, leading to increased glycoeogenesis, reduced glucose consumption, and glycogen synthesis in the liver, which is a major manifestation of insulin resistance in the liver (Haemmerle et al., 2006). In this study, the model of NAFLD was used *in vivo* to further investigate the effect of HCQ on liver glucose and lipid metabolism and insulin sensitivity (Chakrabarti et al., 2009). Insulin resistance and NAFLD are highly correlated comorbidities. Fatty liver can show different clinical symptoms under different physiological conditions, including hyperglycemia, hyperlipemia, and insulin resistance. It has been demonstrated by studies that mouse models that have no, or are low in, hepatic lipid accumulation retained insulin sensitivity even if in time of obesity (Samocha-Bonet et al., 2012; Montgomery et al., 2013). Similarly, humans with metabolically normal obese were insulin sensitive with remarkable lower hepatic lipid accumulation compared with obese but insulin-resistant individuals (Takahashi et al., 2012). Thus, concluding the pathologic mechanisms that result in lipid deposition in the liver is more crucial when it also leads to insulin resistance and diabetes mellitus. In the light of the most prevalent and widespread “two-hit” model for NAFLD, when the “first hit” involves fat accumulation (involved cholesterol and TG) in hepatic cells (Schuster et al., 2018), “second hit” is succeeding hepatic injury, inflammation, and fibrosis. The characteristics of obesity-induced inflammation involve the production and elevated expression of pro-inflammatory factors and the activation of relevant pathways, such as the NF-κB pathway. Pro-inflammatory factors are found in liver, containing acute-phase cytokines, such as TNF-α, interleukin (IL)-1β, and chemokines, including monocyte chemoattractant proteins (MCPs) (Geisler and Renquist, 2017). Corresponding to this theory, the present NAFLD models involved in HFD feeding led to the remarkable upregulation of inflammatory response and lipid accumulation. In the meantime, HCQ significantly decreased the hepatic steatosis of HFD mice, indicating that HCQ performs an important function in preventing the development of NAFLD (**Figure 4A–D**). Next, the expression changes of genes referring to inflammatory response of liver tissue were investigated. The expression rates of macrophage-specific genes (Cd68: a pro-inflammatory M1 phenotype marker; Arg1: an anti-inflammatory M2 phenotype marker) and pro-inflammatory genes (IL-1β, MCP1 and TNF-α) were examined by quantitative RT-PCR. These results revealed that HCQ could reduce excessive inflammatory response in obese mice (**Figure 4E**). In addition, HCQ markedly promoted the glucose uptake in HepG2 cells, which was consistent with the *in vivo* experiments, and this effect became apparent as the concentration and incubation time increased (**Figure 4F**). These results suggest that HCQ improved hepatic lipotoxicity and insulin resistance.

In addition to excessive FFAs, which deposit in the liver and increase its lipid content rapidly, the increase of DNL can also lead to hepatic steatosis (Iizuka et al., 2004). Under physiological conditions, elevated blood glucose level can up-regulate the expression of lipase and promote the storage of liver fat. There are two main signaling pathways: one is direct activation of carbohydrate response element binding protein (ChREBP), and the other is stimulation of steroid response element binding protein 1c (SREBP1c) signaling pathway by increasing blood insulin levels (Deng et al., 2012). The up-regulation of SREBP1c and CHREBP can further promote the expression of acetyl coenzyme A carboxylase (ACC) and fatty acid synthase (FAS). It has been proved that hyperinsulinemia can increase the expression of SREBP1c and CHREBP and can further promote the occurrence of hepatic DNL (Schwarz et al., 2003; Ido-Kitamura et al., 2012). The incidence of liver DNL in hyperinsulinemic obese patients after high fat diet was significantly higher than that in normal insulin obese or lean weight patients (Brun et al., 1996). Next, the expression changes of the involved DNL genes (SREBP1c and ChREBP) and lipogenic enzymes (FAS and ACC) were examined. The present observation demonstrated that HCQ decreased the expression of DNL genes and protein-downregulated lipogenic enzyme activity in liver tissues, which had a hepatoprotective effect (**Figure 6**).

PPARγ, a ligand-activated orphan nuclear receptor, plays a key role in regulating glycolipid metabolism. Fatty acids have been shown to be important ligands. PPARγ is a key transcription factor of anabolism and plays an important role in lipid synthesis. Under HFD conditions, PPARγ is directly bound to PPARγ response elements, regulating genes related to fat synthesis and glucose homeostasis (Matsusue et al., 2003; Imai et al., 2004; Tontonoz and Spiegelman, 2008). However, the mechanism on how HCQ regulates PPARγ-dependent lipogenesis remains unclear. In fatty liver disease, the increase of hepatic DNL is thought to be one of the mechanisms responsible for lowering hyperglycemia, while the increase of β-oxidation is associated with better energy expenditure and reduced lipid toxicity. The increased expression of genes, including PPAR, was the result of chronic lipid accumulation in the liver, further inducing an increase in DNL in fatty liver disease. The onset of this vicious metabolic cycle suggests that hepatocytes are following the metabolic characteristics of fat cells to adapt to long-term fat accumulation (Geisler and Renquist, 2017). In the present study, HCQ decreased the mRNA and protein expression of PPARγ, but increased the expression of beta-oxidation genes carnitine palmitoyltransferase I (CPT1α) and CPT1β in HFD mice (**Figure 6**). These results indicate that HCQ improved insulin resistance and hepatic lipotoxicity by decreasing adipogenesis through the PPARγ pathway.

In summary, the data presented in the present study clearly show the inhibitory effect of HCQ on HFD-related obesity and its related metabolic complications. HCQ affects HFD-related sDNL through the PPARγ signaling pathway in the liver. In obese mice, HCQ treatment improved hyperinsulinemia, alleviated hepatic steatosis, and induced weight loss. In conclusion, based on the abovementioned evidence, HCQ is a potential drug that can improve metabolic abnormalities and can thus reduce diet-related obesity.

## Data Availability

The raw data supporting the conclusions of this manuscript will be made available by the authors, without undue reservation, to any qualified researcher.

## Ethics Statement

After the review by the ethics committee of Ruijin Hospital, its research content and scheme design basically conform to the ethical norms.

## Author Contributions

X-BC and C-DY contributed to the conception and design. X-BC, XQ, Y-TS, and YS contributed to the acquisition and assembly of data. X-BC, XQ, RN, J-LT, J-NY, and HS contributed to the analysis and interpretation of the data. X-BC, XQ, and RN contributed to the drafting of the article. All authors contributed to the critical revision of the article for important intellectual content. Final approval of the article was given by all authors.

## Funding

This project is supported by the National Natural Science Foundation of China (81801596), co-founded by Shanghai Sailing Program (18YF1414500).

## Conflict of Interest Statement

RN was employed by Shanghai Pharmaceutical Medicine.

The remaining authors declare that the research was conducted in the absence of any commercial or financial relationships that could be construed as a potential conflict of interest.

## Abbreviations

HCQ, Hydroxychloroquine; NAFLD, nonalcoholic fatty liver disease; SLE, systemic lupus erythematosus; ND, normal chow diet; HFD, high fat diet; FBG, fasting blood glucose; IPGTT, intraperitoneal glucose tolerance tests; ITT, insulin tolerance test; AUC, areas under the curve; IRT, insulin release test; HOMA, homeostasis model assessment; HOMA-IR, insulin resistance index; HOMA-IS, insulin sensitive index; HOMA-β, basic function of pancreatic β cell; TG, triglyceride; FFA, free fatty acids; TC, total cholesterol; LDL-c, low density lipoprotein-cholesterol; HDL-c, high density lipoprotein-cholesterol; GAPDH, glyceraldehyde phosphate dehydrogenase; PPARγ, peroxisome proliferator-activated receptor gamma; Mgat-1, monoacylglycerol O-acyltransferase; SREBP1c, sterol regulatory element-binding transcription factor 1; ChREBP, carbohydrate response element binding protein; FAS, fatty acid synthetase; ACC, acetyl-coa carboxylase; p-AKT, phosphorylated of serine/threonine-specific protein kinase; t-AKT, total of serine/threonine-specific protein kinase; p-IRS1, phosphorylated-insulin receptor substrate 1; t-IRS1, total-insulin receptor substrate 1; H&E, hematoxylin and eosin; S.E.M., standard error of measurement; ELISA, enzyme-linked immunosorbent assay; IL-1β, interleukin-1β; TNFα, tumor necrosis factor α; CD68, cluster of differentiation 68; MCP-1, monocyte chemoattractant proteins 1; Arg-1, arginase-1; CPT1, carnitine palmitoyltransferase I; DNL, *de novo* lipogenesis.
